# Back to EGF+61 genetic polymorphisms and lung cancer risk: looking to the future!

**DOI:** 10.1590/S1516-31802012000600011

**Published:** 2013-01-18

**Authors:** Ramon Andrade De Mello

**Affiliations:** I MD. Medical Oncology Resident in the Department of Medical Oncology, Portuguese Oncology Institute, Porto, Portugal; and Assistant Professor in the School of Medicine, Department of Medicine, University of Porto, Porto, Portugal.

Dear Editor,

Recently, studies in the field of epidermal growth factor (*EGF) +61A/G* polymorphisms have shown their relevance in relation to cancer behavior. The role of *EGF+61* genotypes has been determined in relation to several types of cancer, such as gliomas, gastric cancer and colorectal cancer (CRC). In 2011, it was demonstrated that these polymorphisms could also serve as predictive biomarkers for CRC patients treated with cetuximab.^1^ In relation to lung cancer, controversy continues to surround the results.^2^^,^^3^^,^^4^ Thus, the purpose of this letter is to provide a point of view and discuss relevant issues within this framework, focusing on a Portuguese study^4^ that contrasts with previous Asian studies^2^^,^^3^ published in the literature.

## *EGF+61* POLYMORPHISM AND LUNG CANCER

Since the last decade, *EGF+61A/G* polymorphisms have been studied as a risk factor for cancer.^5^ The first article published showing this association was on malignant melanoma.^5^ In this work, the authors also suggested that higher serum EGF expression could be biologically explained by the proximity of the +61G locus to a region involved in *EGF* gene regulation. In September 2011, at the European Multidisciplinary Cancer Congress, in Stockholm, the association between *EGF+61A/G* polymorphisms and the risk of non-small-cell lung cancer (NSCLC) was shown for the first time in a Portuguese population.^4^ However, the Portuguese study was discordant with the findings of Kang et al.,^3^ who did not report this association in relation to overall lung cancer. Certain concerns could explain these differences between the results, such as ethnic divergences. According to a recent meta-analysis,^5^ presence of the +61G allele is indeed considered to be a key point in the steps towards carcinogenesis because of its property of increasing serum EGF and thereby stimulating proliferation, angiogenesis and metastasis.^5^ This interaction between serum EGF and its receptor (EGFR) is very important within the NSCLC framework. It induces tumor aggressiveness, especially regarding four pathways: 1) Phospholipase Cg (PCL-g); 2) Phosphatidylinositol 3-kinase (PI3K); 3) Signal transducer and activator of transcriptions (STATS); and 4) Ras, Raf, MEK, ERK, MAPK (mitogen-activated protein kinase).^1^^,^^5^ Nevertheless, another Korean study conducted by Lim et al.^2^ has shown a relationship between *EGF*+*61A/G* polymorphisms and lung cancer.

## CURRENT AND FUTURE DIRECTIONS

Currently, EGFR mutation is among the main tools available to medical oncologists in clinical practice, for improving NSCLC patients’ care. Targeted therapies against these mainly molecular pathways and novel biomarkers are objects of primary interest. As occurred in relation to colorectal cancer (CRC), it was suggested that *EGF+61A/G* polymorphisms might be involved in NSCLC susceptibility. Subsequently, it was demonstrated that *EGF+61A/G* polymorphisms could also serve as predictive biomarkers for CRC patients treated with cetuximab.^1^ We believe that this is the key point in *EGF+61A/G* polymorphism research within the lung cancer context. First, determine whether *EGF+61A/G* polymorphisms with NSCLC susceptibility are present. Second, test their influence on patient outcomes and assess whether they could serve both as risk markers and as predictive biomarkers. Finally, assess their role in cancer prevention, perhaps in correlations with other risk factors and as contributions towards creating nomograms in order to predict cancer risk. This could help in future public preventive healthcare strategies, but great effort is still required in order to validate these issues in other populations.
